# Response of Chlorophyll Fluorescence Characteristics and Dissolved Organic Matter for Marine Diatom *Skeletonema dohrnii* under Stress from Penicillin and Zn^2+^

**DOI:** 10.3390/plants10122684

**Published:** 2021-12-07

**Authors:** Yang Liu, Xiaofang Liu, Jun Sun

**Affiliations:** 1 Institute of Marine Science and Technology, Shandong University, Qingdao 266237, China; youngliu@mail.sdu.edu.cn; 2 College of Marine Science and Technology, China University of Geosciences (Wuhan), Wuhan 430074, China; liuxiaofang425@163.com

**Keywords:** chlorophyll fluorescence variables, DOM, fluorescence indices, fluorescence peaks

## Abstract

*Skeletonema dohrnii* is a good model diatom for studying environmental stress and has promising applications and prospects in various fields. Antibiotics and heavy metals are commonly exceeded in the nearshore marine habitats. In this work, we investigated the effects of an antibiotic (penicillin, 2 µg/L) and a heavy metal ion (Zn^2+^, 10 µmol/L) stress on marine diatom *S. dohrnii*, mainly using excitation-emission matrices (EEMs) fluorescence methods and OJIP test. Results indicated that algal cells grown with the antibiotic showed higher biomass, specific growth rate, doubling time, chlorophyll *a*, and chlorophyll fluorescence variables. Moreover, excess zinc had negative effects on *S. dohrnii*. We found that zinc not only inhibited the relative photosynthetic electron transfer efficiency but also reduced the Chl *a* content, which ultimately affected algal growth and organic matter production. In addition, the combined effect of penicillin and Zn^2+^ further affected the physiological state of *S. dohrnii*. The dissolved organic matter (DOM) characteristics of the four cultures were also different, including fluorescence indices (fluorescence index, biological index, β/α, and humification index) and fluorescence peaks (peaks A, C, M and T). In brief, characterization of chlorophyll fluorescence characteristics and DOM-related variables are important for understanding the effects of environmental stress on microalgae.

## 1. Introduction

Phytoplankton (including eukaryotic and prokaryotic cells) play an important role in the ecosystem as the main primary producers in the ocean [1]. They play an irreplaceable role in the balance and stability of marine ecology. DOM is a complex mixture of organic substances in aquatic environments (such as water, soil, and sediment), including protein-like substances, humic-like substances and other aliphatic/aromatic organic compounds [2]. Additionally, DOM plays a vital role in natural aquatic systems and geochemical/biochemical processes [3,4,5]. Antibiotics and metal ions pollution are a serious environmental problem. They can enter organisms through a variety of ways (e.g., water, soil, and food chain, etc.) in the ecosystem and exacerbate the harm to the environment. Moreover, antibiotics can combine with metal ions and affect the chemical behavior of microalgae in the oceanic environment [6]. Recently, excitation–emission matrices (EEMs) fluorescence spectroscopy has been used as a powerful tool to quantify DOM in a wide range of environmental detection because of its sensitivity, fast response and high selectivity [7,8]. Equally important, OJIP-test-derived variables obtained automatically via PAM fluorometer have been widely used in algae monitoring [9]. More recently, there has been growing recognition of the vital links between environmental pollutants (antibiotics or metal ions) and the physical or chemical properties of the algae.

The diatom *Skeletonema* spp. are widely distributed throughout the ocean in habitats spanning a full range of observed area. Meanwhile, it is easily cultured in the laboratory, and each species within the same genus has its own characteristic behavior curve [10], reflecting physical and chemical characteristics changes in adaptation to its environmental circumstances [11]. Most investigations evaluate various environmental factors influence directly or indirectly under different conditions, including illumination, temperature, salinity, CO_2_, nutrients, and pH [12]. Photosynthesis is one of the most sensitive physiological processes in microalgae, and stress by environmental factors mainly destroys photosystem II (PS II). The destruction of PS II by stress can be reflected by the changes in chlorophyll fluorescence variables. Results from earlier studies demonstrate that the addition of antibiotics can have biological effects on microalgae, and that heavy metal stress can also affect the growth and chlorophyll fluorescence properties of microalgae. Evidence from a number of experimental studies has established that *Skeletonema dohrnii* is highly resistant and has been used extensively in the study of environmental stresses [13]. The fate of diatoms in a changing water environment is uncertain due to the potentially different responses of each species. To date, there is a relative paucity of empirical research focusing specifically on effects of pollution (antibiotics and metal ions) on algal photosynthetic activity and dissolved organic matter (DOM).

In this study, *S. dohrnii* was cultured in batch mode. Subsequently, we established different treatments to investigate: (1) how the addition of antibiotics and heavy metals affects the photosynthetic activity of diatoms; and (2) also to understand *S. dohrnii* changes in DOM under stress conditions (2 µg/L penicillin and 10 µmol/L Zn^2+^). Characterization of chlorophyll fluorescence and DOM associated variables were important for our increased understanding of the effects of penicillin and Zn^2+^ in marine diatoms.

## 2. Material and Methods

### 2.1. Microalgae Culture Conditions, Growth, and Experimental Set-Up

The marine diatom *S. dohrnii* was originally isolated from the central Yellow Sea, China [14], and subsequently identified and preserved in our laboratory. The cells of *S. dohrnii* were cultured in transparent conical flasks with artificial seawater (ASW) medium [15] at 25 °C under a 14 h:10 h light:dark cycle with a light intensity of 100 μmolphotons μmol m^−2^ s^−1^ and shaking at 160 rpm on a shaker (HY-8, Changzhou Guohua Electric Appliance Co., Ltd., Changzhou, China). Cultures were maintained under these conditions for approximately 30 days before the initiation of the experiment.

*S. dohrnii* cells were collected via centrifugation (5000 rpm, 5 min) after cultivation in ASW medium. The cells were washed with Milli-Q water and centrifuged a second time at 5000 rpm for 5 min. Then, algal cells were inoculated in 2 L flasks containing 1.5 L ASW medium. The initial biomass concentration of the *S. dohrnii* was about (2.03 ± 0.02) × 10^8^ cells/L. We chose penicillin (2 µg/L), Zn^2+^ (10 µmol/L) and penicillin (2 µg/L) + Zn^2+^ (10 µmol/L) to represent the single and combination experiments, respectively. For simplicity, we named blank (*S. dohrnii*-only, Blank) and different treatments (*S. dohrnii* with penicillin, SA; with Zn^2+^, SZ; with penicillin and Zn^2+^, SAZ). All experiments were carried out in triplicate.

### 2.2. Determination of Microalgal Growth and Chl a Content

Algal cells were cultivated at a constant temperature of 25 °C in a shaker at a shaking rate of 160 rpm min^−1^. To regularly monitor the algal growth, 100 μL of microalgae solution was collected onto the blood cell counting chamber and microalgal cells were counted immediately using an inverted microscope (Olympus BX51). Specific growth rate and doubling time of *S. dohrnii* cultured in the laboratory were calculated based on the following equations:

Specific growth rate (μ,/d) = (ln N_a_ − ln N_b_)/(t_a_ − t_b_)

Doubling time (T_d_, t) = ln 2/μ

where N_a_ and N_b_ denote the algal abundance (cells/L) at times t_a_ and t_b_, respectively.

The content of Chl *a* was determined by referring to Mera et al. [16] with a few adjustments. The absorbance of the algal solution (supernatant) at 649 nm and 665 nm was measured via UV-spectrophotometer (T2600, China) with 95% alcohol (*v/v*) as blank control. The following formula was used to calculate the concentration of Chl *a*:

Chl *a* = 13.95 (A665) − 6.88 (A649).

### 2.3. Determination of Chlorophyll Fluorescence

The algal solution had 20 min of dark acclimation at 26 °C. The fluorescence parameter (Fix area, quantum yield, QY; potential photochemical efficiency of photosystem II, F_v_/F_m_; potential activity of photosystem II, F_v_/F_o_; and total light energy flux, Pi_Abs) of the chlorophyll was determined concurring to Markou et al. [17], utilizing the AquaPen AP110/C (Drassov, Czech Republic).

### 2.4. EEMs Fluorescence Spectroscopy

Artificial seawater samples for DOM analyzed from 10 mL samples filtered through 0.7 µm combusted GF/F filter (25 mm diameter) onto pre-combusted (450 °C for 6 h) glass vials. Even though these filters have a larger pore size, as compared to 0.2 µm polycarbonate filters, they have advantages that demonstrated their application in this research [18]. Then, the EEMs spectra of the DOM were measured with F-7100 fluorescence spectrophotometer (Tokyo, Japan). The voltage of photomultiplier tube (PMT) was set to 700 V. Fluorescence spectra detected subsequent scanning of excitation (Ex) from 200 to 450 nm and emission (Em) from 250 to 550 nm. Ex and Em slits were maintained at 5 nm and the scanning speed was set at 12,000 nm/min.

Instrument corrections were performed according to the procedure recommended by the Hitachi F-7100 instruction manual. Blank subtraction using ultrapure water (Milli-Q) was performed for each DOM spectrum in order to remove most of the Raman scatter. The correction was followed by Raman calibration according to the literature [19]. Fluorescence information for all data was used to assess using water Raman units (R.U.). Fluorescence indices were further derived from the EEMs: fluorescence index (FI) [20], biological index (BIX) [21], β/α (a ratio of two known fluorescing components [21], and humification index (HIX) [22].

### 2.5. Elemental Stoichiometry Analysis

The dissolved organic carbon (DOC) was measured using a total organic carbon analyzer (TOC-3100, Jena, Germany). To determine the particulate organic carbon (POC) and particulate organic nitrogen (PON) in *S. dohrnii*, cells were harvested using pre-combusted (450 °C for 6 h) GF/F glass fiber filters (25 mm diameter). The samples were acidified with 200 μL of 0.2 N HCl and dried overnight in an oven at 60 °C before analysis with a Costech Elemental Analyzer (ECS4010, Milan, Italy)

### 2.6. Statistical Analysis

In this study, Statistical analysis of all data was performed with unpaired *t*-test analysis using SPSS software (version 26.0). Triplicate samples of each condition were taken into consideration, and comparison was done between groups of cells grown at penicillin and Zn^2+^. The correlation analysis and chromophoric dissolved organic matter (CDOM) associated variables were obtained using the “ggplot2”, “corrplot”, “RColorBrewer” and “reshape2” package in RStudio software. The measured data were reported as mean ± standard deviation (SD). The value of *p* < 0.05 was considered a statistically significant difference.

## 3. Results

### 3.1. Growth Profiles of S. dohrnii

**Figure 1:**
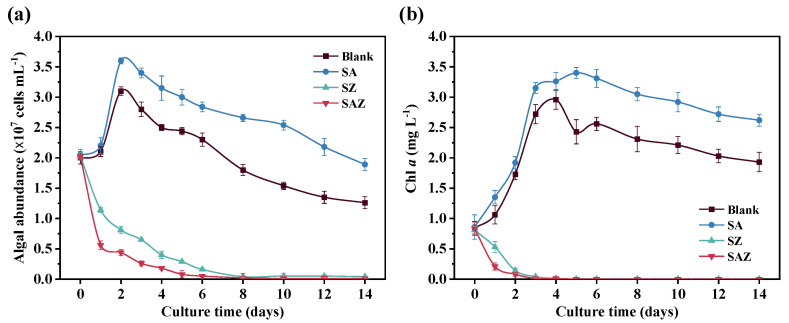
Growth curves (**a**) and Chl *a* concentration (**b**) of *S. dohrnii* with different treatment in artificial seawater. Blank, *S. dohrnii* only; SA, with penicillin for *S. dohrnii*; SZ, with Zn^2+^ for *S. dohrnii*; SAZ, with penicillin and Zn^2+^ for *S. dohrnii*. Each value represents mean ± SD (*n* = 3). Note error bars are smaller than the symbol sizes in some cases.

*S. dohrnii* was cultivated in artificial seawater without the lag phase (Figure 1a). After 2 days of cultivation, *S. dohrnii* had the highest algal abundance of (3.10 ± 0.07) × 10^8^ (Blank) and (3.60 ± 0.05) × 10^8^ cells/L (SA), respectively (Figure 1a). The algal abundance of *S. dohrnii* significantly decreased from around (2.03 ± 0.05) × 10^8^ (SZ) to (0.04 ± 0.01) × 10^8^ and (0.02 ± 0.01) × 10^8^ cells/L (SAZ), respectively, after 8 days of cultivation. In other words, the cells concentration in the SZ and SAZ treatments decreased from day 0 and approached zero at day 8 (Figure 1a). As listed in Table 1, specific growth rates for each of treatment were (0.389 ± 0.006) d^−1^ for Blank, and (0.492 ± 0.004) d^−1^ for SA. The doubling times were (1.779 ± 0.011) d and (1.407 ± 0.007) d for Blank and SA. There was no significant difference between both groups. The specific growth rates and doubling times of SZ and SAZ showed a negative increasing trend, so they are not shown in Table 1. The effects of antibiotics and Zn^2+^ on the Chl *a* content of *S. dohrnii* are shown in Figure 1b. In the whole culture times, there were significant differences among four cultures. The Chl *a* content of *S. dohrnii* also indicated that SA grew best in artificial seawater compared with the other cultures (Blank, SZ, and SAZ) (Figure 1b).

### 3.2. Chlorophyll Fluorescence

Photosynthesis is one of the major metabolic activities of algae cultivated in the natural environment. Nevertheless, chlorophyll fluorescence of cells was analyzed to assess the growth of *S. dohrnii* in response to various treatments in artificial seawater.

The effects of different treatments (penicillin and Zn^2+^) on the photosynthetic property of *S. dohrnii* were evaluated by monitoring the chlorophyll fluorescence characteristics. For these fluorescence variables (i.e., fix area, QY, F_v_/F_m_, and F_v_/F_o_), there was a significant increase in Blank and SA during the first two days, followed by a general decrease. SZ and SAZ gradually decreased and tended to zero throughout the culture time. Moreover, we found that Blank was generally higher than the other treatments in variables of QY, F_v_/F_m_, and F_v_/F_o_, except for the fix area. This suggests that the addition of penicillin and Zn^2+^ affected the chlorophyll fluorescence properties of *S. dohrnii*.

**Figure 2:**
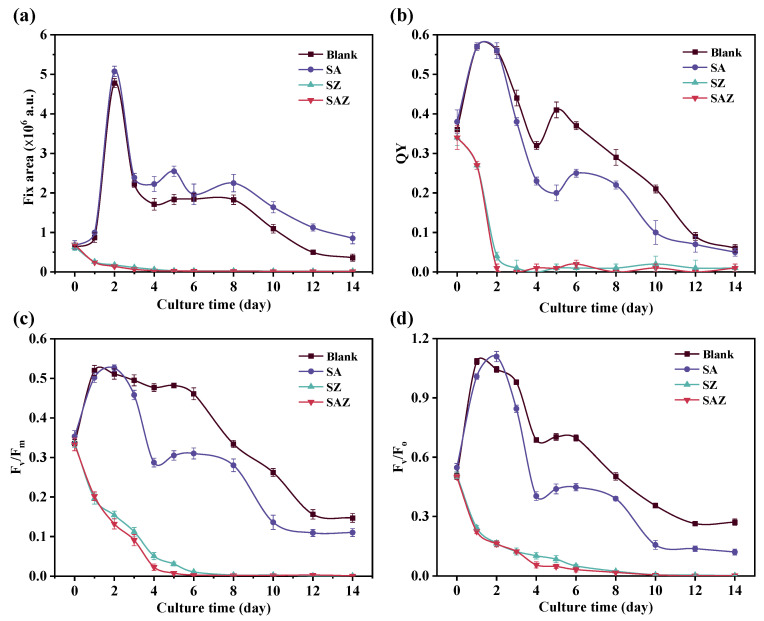
Chlorophyll fluorescence characteristics of *S. dohrnii* with different treatment in artificial seawater. Blank, *S. dohrnii* only; SA, with penicillin for *S. dohrnii*; SZ, with Zn^2+^ for *S. dohrnii*; SAZ, with penicillin and Zn^2+^ for *S. dohrnii*. Each value represents mean ± SD (*n* = 3). Note error bars are smaller than the symbol sizes in some cases.

As shown in Figure 2, a higher maximum PS II efficiency activity was found in SA than the other three conditions (Blank, SZ, and SAZ), meanwhile, values of fix area, QY, F_v_/F_m_, and F_v_/F_o_ significantly decreased at the end of cultivation (*p* < 0.05). Looking at Figure 2a, it is apparent that the fix area changes trend significantly with Blank and SA in the whole culture stage compared with SZ and SAZ. In addition, QY, F_v_/F_m_, F_v_/F_o_ had similar trends, and the differences among treatments were also more significant (*p* < 0.05, Figure 2b–d).

### 3.3. DOC, POC, PON Concentrations, and POC/PON

There were significant differences in carbon and nitrogen concentrations between the four cultures (Table 2). The carbon concentration of the four cultures increased on day 14. The largest increase occurred in DOC, increasing between day 0 and day 14 by 0.189 mg/L for SA. The highest POC valus of SA were in the degradation phase, while the POC values of SAZ were reduced compared with the other cultures. In addition, for PON values, there was a general increase between day 0 and day 14, with an increase of 0.040 mg/L for Blank, 0.032 mg/L for SA, 0.011 mg/L for SZ, and 0.001 mg/L for SAZ. However, the POC/PON increased in all culture groups except for the SAZ, where it decreased, and the highest increase was observed in the SA.

### 3.4. Fluorescence Characteristics

**Figure 3:**
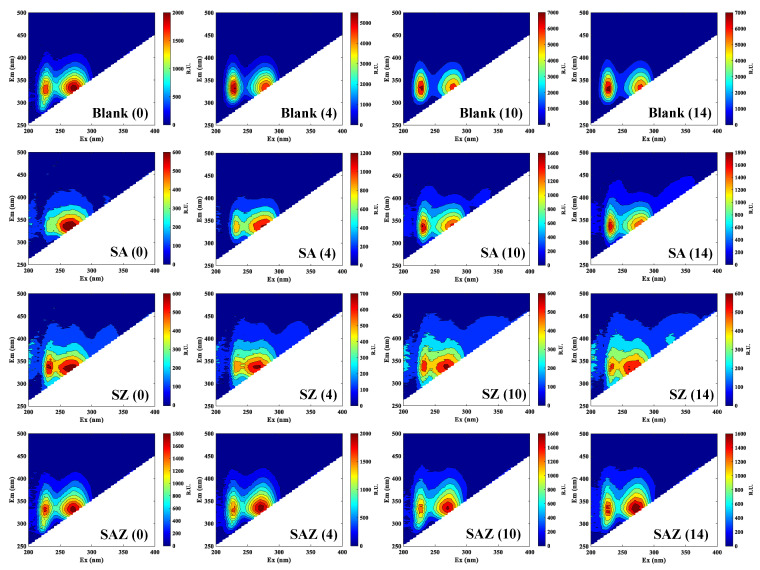
EEMs of DOM fluorescence for *S. dohrnii* with different treatment at four time point. Note that different color bars are used for each group (Raman units, R.U.). Blank, *S. dohrnii*-only; SA, with penicillin for *S. dohrnii*; SZ, with Zn^2+^ for *S. dohrnii*; SAZ, with penicillin and Zn^2+^ for *S. dohrnii*. (0), (4), (10), and (14) are represented as 0, 4, 10, and 14 days, respectively.

The fluorescence patterns were similar between all treatment groups, with two fluorophores (peak T) being the most obvious (Figure 3, Table 3). The peak patterns of remained consistent throughout the culture cultivation period. The SA exhibited a single-peak pattern on day 0, and the overall peak pattern shifted to longer wavelengths with increasing culture time. A similar situation occurred in the SZ, unstructured fluorescence with slightly enhanced fluorescence appears around peak T. The SZ essentially formed a three-peak pattern on day 14.

**Figure 4:**
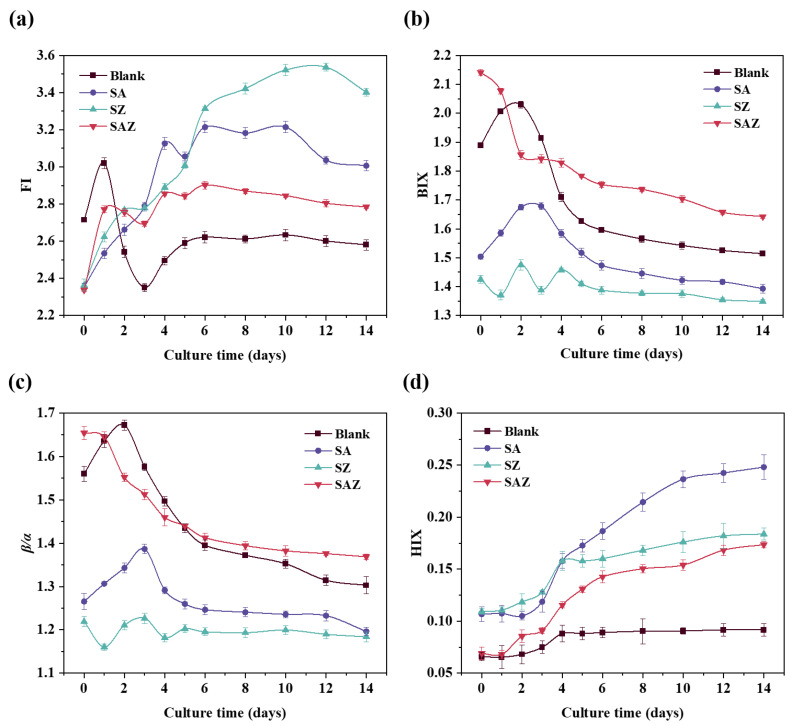
Changes in fluorescence indices of *S.dohrnii* of different treatments during the culture time. (**a**–**d**) are represented as FI, BIX, β/α, and HIX, respectively. Error bars represent the standard error for duplicate cultures. Note error bars are smaller than the symbol sizes in some cases.

The fluorescence indices were further used as variables indicating changes in fluorescence characteristics under the different treatments of *S. dohrnii*. From Figure 4, we can see the different trends of fluorescence indices for each treatment. The FI and HIX showed overall an increasing trend, except for FI in Blank. In contrast, BIX and β/α generally showed a decreasing trend, moreover, the values of Blank and SA were higher than treatments of SZ and SAZ.

**Figure 5:**
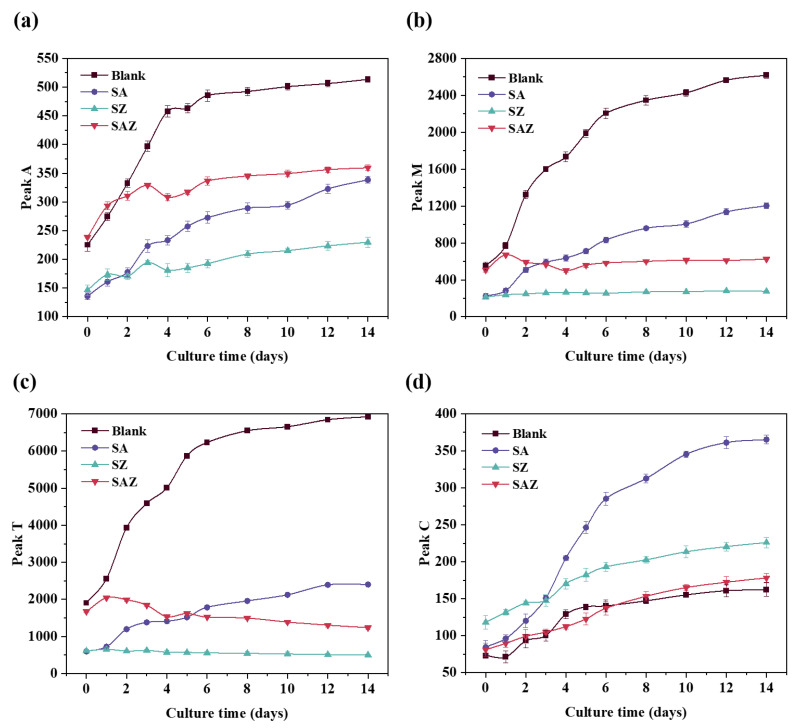
Changes in fluorescence peaks of *S. dohrnii* in the different treatments during the culture time. (**a**–**d**) are represented as peaks A, M, T, and C, respectively. Error bars represent the standard error for duplicate cultures. Note error bars are smaller than the symbol sizes in some cases.

Generally, the fluorescence intensity changes of the peaks (A, C, M, and T) were basically similar, which increased with increasing culture time (Figure 5). The fluorescence intensity of SZ was higher in peaks A and M than the others. For peaks (A, M, and T), Blank had the highest value and generallyhad a higher value than others. Additionally, for peak C, the value of SA was generally higher compared to the other treatments. In addition, there was no significant change in SZ and SAZ during the culture time (Figure 5b,c). The SAZ had a brief increase on the first day, then became flat or decreased (Figure 5b,c).

## 4. Discussion

### 4.1. Growth Conditions of S. dohrnii under Different Treatments

When antibiotic stress is applied, it is well recognized that antibiotics inhibit the growth of bacteria and usually also influence the growth of algae. In addition, antibiotics might be altered the *S. dohrnii*-associated bacterial community diversity. It was noteworthy that the antibiotic used in this study was penicillin at a concentration of 2 µg/L. The increase in the number of cells in *S. dohrnii* implies that the cause may be due to the antibiotics removing bacteria that are harmful to the growth of the algae [27,28]. One possible explanation might be due to a lack of substrate competition from fast-growing bacteria, or release of the pressure from bacterial degradation. However, most previous studies reported that antibiotics, such as erythromycin and chloramphenicol, can inhibit the growth of algae [29,30]. Additionally, higher concentration of antibiotics had a direct adverse effect on the growth of algae like florfenicol, furazolidone, and above-mentioned antibiotics. The concentration and type of antibiotics were both important factors. Several studies have shown that Chl *a* in algal cells is also affected to some extent when the algae are stressed. As the concentration of heavy metal ions increases, Chl *a* suffers more negative effects. The accumulation of reactive oxygen species damages the algal cell structure and limits the synthesis of Chl *a*. However, based on our observation, the values of algal abundance, Chl *a*, and chlorophyll fluorescence characteristics were different from the other treatment groups (Figure 1 and Figure 2). Previous studies have concluded that antibiotics and heavy metal ions affect the growth or specific metabolic pathways of eukaryotes to varying degrees. Together, we could suggest that penicillin (2 µg/L) and Zn^2+^ (10 µmol/L) indirectly affect the physiological state of *S. dohrnii*.

### 4.2. Chlorophyll Fluorescence Variables of S. dohrnii under Different Treatments

Photosynthetic activity parameters are widely used to describe the photosynthetic characteristics of algal cells [31]. Besides, photosynthetic activity parameters provide a comprehensive understanding of the performance variables of microalgal cell photosystems under antibiotic and heavy metal ion stress, the specific processes of the toxic effects of pollutants on microalgae still need more penetrating studies. F_v_/F_m_ is the main parameter that measures photosynthetic performance and is an important index that directly expresses the photosynthetic efficiency of microalgae. It has been suggested that the stress-induced decrease in F_v_/F_m_ is a short-term response of the photosystem under stress conditions, which may be a reconstructed equilibrium between light energy uptake and utilization by microalgae, minimizing damage to the algae [32]. Several studies have shown that the photosynthetic efficiency of algae decreases when subjected to environmental stress [33,34]. In this study, the F_v_/F_m_ and F_v_/F_o_ ratios of the algae decreased rapidly with increasing incubation time, indicating that the photosynthetic system of the microalgae was inhibited due to the addition of Zn^2+^. This may be due to the fact that heavy metal ions blocked photosynthetic electron transfer between q_a_ and q_b_, which in turn formed more q_b_-non-reducing PS II reaction centers and this led to a reduction in oxygen evolution from PS II reaction centers. In this study, we found that the variation in F_v_/F_m_ and F_v_/F_o_ ratios were different for all cultures, suggesting that penicillin (2 µg/L) and Zn^2+^ (10 µmol/L) impact photosynthetic electron transport in algal cells.

### 4.3. Analysis of Fluorescence Characteristics

**Figure 6:**
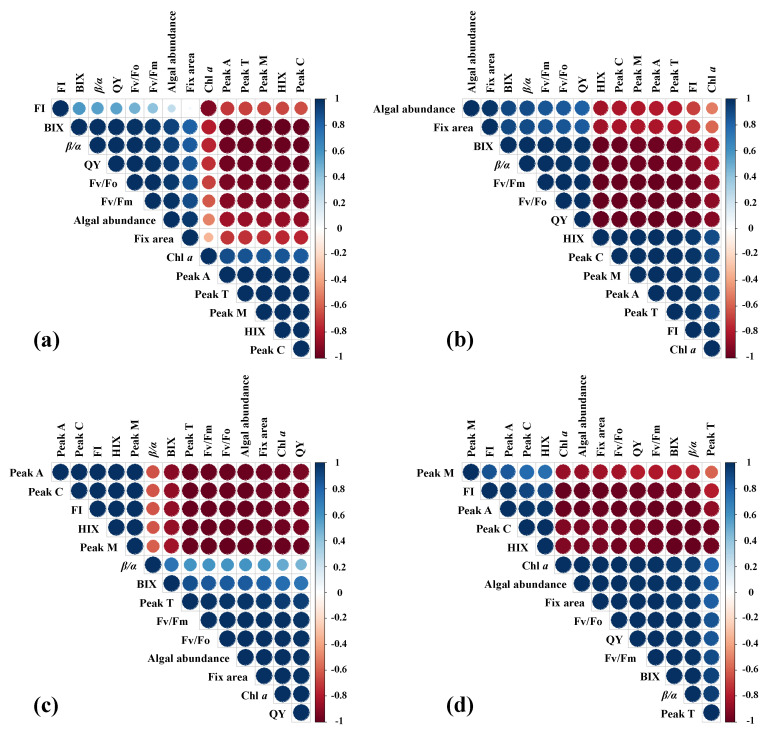
Correlation analysis between different variables of *S. dohrnii* with different treatment in artificial seawater. (**a**–**d**) are represented as Blank, SA, SZ, and SAZ, respectively. Note the color and size of the pie indicate the correlation coefficient. The blue color indicates positive correlation and red color indicates negative correlation.

Correlation analysis showed significant variability between the responses of penicillin (2 µg/L) and Zn^2+^ (10 µmol/L) addition to the different treatments (Figure 6). Moreover, strong correlations were found between the variables and indices (Figure 6). The Chl *a* was negatively correlated with the algal abundance, which positively correlated with the chlorophyll fluorescence variables (i.e., fix area, QY, F_v_/F_m_, and F_v_/F_o_) and fluorescence peaks (i.e., peaks A, M, T, and C) in Blank and SA (Figure 6a,b). However, there are generally opposite results for SA and SAZ (Figure 6c,d). These correlations indicated that Chl *a* can be used as a factor indicating the growth status of microalgae. In this study, the reduction of photosynthesis could be well associated with the change of PS II function in excess Zn^2+^ concentration. More generally, zinc is indispensable for electron transport in photosynthesis and by various enzyme systems as an essential element for metabolic and physiological processes. Numerous studies suggested that inhibition of photosynthesis by metal ions had been observed in algae [35,36]. As the concentration of Zn^2+^ exceeded 1 µmol/L, the growth of *Scenedesmus subspicatus* and *Chlamydomonas reinhardtii* was inhibited [37]. Once concentration exceeded 5 µmol/L, the relative electron transport rate and photochemical efficiency of algal cells were also seriously inhibited [38]. However, Zn^2+^ stress at 10 µmol/L not only inhibited the relative photosynthetic electron transfer efficiency, but also reduced the functionality of mitochondrial membranes and Chl *a* content, which ultimately affected algal growth and organic matter production [39].

Some laboratory experiments have depended on additions of carbon or high nutrients substrates to investigate DOM formation by microalgae or bacteria [40]. In this study, our maximum DOM fluorescence intensity for all treatments (SA, SZ, and SAZ) was low, except for the Blank; less than fluorescence intensities in several laboratory experiments [40]. This phenomenon is most likely due to the addition of penicillin and Zn^2+^, which affect the production and fluorescence intensity of diatom *S.dohrnii*-derived DOM. In addition, it may also be related to the species of microalgae or culture medium. Taken together, our previous study and results suggest that the fluorescence intensities generated in microalgae culture experiments are relatively low when the variability of the instrument is taken into account. Indeed, background fluorescence is helpful for monitoring the low DOM fluorescence by microalgae.

All four cultures produced a pattern of two peaks that were dominated by DOM fluorophores, which suggests different compounds (e.g., aromatic amino acids). This two-peak pattern has been previously demonstrated in monocultures and oceanic origin [41]. The peak is similar to a protein resembles the amino acid tryptophan, and has been confirmed that it plays an important role in the growth of algae [42]. More generally, the differing fluorescence in the region of peak T have been considered as fluorophores present in the amino acid tryptophan. Thus far, a number of studies have reported diffusion in peak T, however, these fluorophores could easily be misinterpreted as humic-like substances, especially in natural samples (freshwater and coastal systems). Since the algal medium in this experiment was an inorganic medium, the observed diffusion of peak T was likely the effect of antibiotics and Zn^2+^ on algae or microbially-transformed planktonic material. A number of recent studies, CDOM generated in culture work on diatoms, prasinophytes, and bacteria showed similar fluorescence patterns to those reported in this study [43]. Although the major fluorophores were similar between individual treatment, the prevalent fluorescence patterns generated overall by algae further supports DOM. However, in previous studies, elevated fluorescence was shown in the region of peak T, which differs from the fluorescence intensity of our study (SZ and SAZ). Taken collectively, these results suggest that penicillin and Zn^2+^ affect the generation of DOM fluorescence peaks. 

The fluorescence index provided a clear insight into the process of DOM changes. The values of FI were all greater than 1.8, indicating that the fluorescence component of OM was mainly produced by microorganisms [44]. The ratio of two known fluorescing components (β/α, where β and α represent more recently derived organic matter and highly decomposed organic matter, respectively). BIX can be used as an indicator of DOM traceability in aquatic ecosystems, and higher values indicated higher degradation of DOM. The significant changes in β/α and BIX values over a short period of time indicated that endogenous carbon products are most likely produced through bacterial processing of DOM. The HIX is a fluorescence index of the degree of organic matter degradation, with higher values characteristic of higher molecular weight and aromatic compounds (i.e., HIX is directly proportional to the humic content of DOM) [45]. The degree of humification gradually intensified with increasing experimental days in all treatments, especially SA. This indicates that the alteration of the algal DOM pool by penicillin and Zn^2+^ led to the change to a more fluorescent component.

## 5. Conclusions

In this work, utilizing combined penicillin and Zn^2+^, the photosynthetic activity and chromophoric dissolved organic matter of *S. dohrnii* were investigated to evaluate the physiological properties and dissolved organic matter characteristics. For the addition of penicillin (2 µg/L) alone, higher values were shown for certain variables compared to other treatments, such as algal abundance, Chl *a*, specific growth rate, DOC, POC, POC/PON, HIX, and peak C. High concentrations of Zn^2+^ (10 µmol/L) can also severely inhibit the electron transfer rate and photochemical efficiency of algal cells. Moreover, Zn^2+^ plays a dominant role in the combined effect of penicillin and Zn^2+^. Since only penicillin (2 µg/L) and Zn^2+^ (10 µmol/L) were selected for this experiment, there were limitations in the choice of pollutant types and concentrations. However, this still provides data to support the investigation of pollutant stress on marine diatoms. In addition, the proliferation of diatoms can cause the initiation of potentially harmful blooms (red tides), and this study also provides a data base for the management of China’s nearshore aquatic environment.

## Figures and Tables

**Table 1:** Specific growth rate and doubling time of *S. dohrnii* with different treatment in artificial seawater.

Treatment Groups	Specific Growth Rate (/d)	Doubling Time (d)
*S. dohrnii* only (Blank)	0.389 ± 0.006 ^a^	1.779 ± 0.011 ^a^
*S. dohrnii* with penicillin (SA)	0.492 ± 0.004 ^b^	1.407 ± 0.007 ^b^

Values within the same row with different letters represent significant difference (*p* < 0.05). Each value represents mean ± SD (*n* = 3).

**Table 2:** Dissolved, particulate organic carbon, particulate organic nitrogen concentrations, and particulate organic carbon to nitrogen ratios for the four different cultures.

Treatment Groups	DOC		POC		PON		POC/PON	
	Day 0	Day 14	Day 0	Day 14	Day 0	Day 14	Day 0	Day 14
Blank	0.368 ± 0.007 ^a^	0.537 ± 0.008 ^a^	0.086 ± 0.005 ^a^	0.394 ± 0.014 ^a^	0.017 ± 0.003 ^a^	0.057 ± 0.011 ^a^	5.059 ± 0.004 ^a^	6.912 ± 0.012 ^a^
SA	0.375 ± 0.006 ^a^	0.564 ± 0.010 ^b^	0.087 ± 0.005 ^a^	0.413 ± 0.011 ^b^	0.016 ± 0.003 ^a^	0.048 ± 0.016 ^a^	5.438 ± 0.004 ^b^	8.604 ± 0.013 ^b^
SZ	0.379 ± 0.011 ^a^	0.423 ± 0.013 ^c^	0.086 ± 0.008 ^b^	0.141 ± 0.017 ^c^	0.018 ± 0.005 ^a^	0.029 ± 0.009 ^a^	4.778 ± 0.005 ^c^	5.035 ± 0.014 ^c^
SAZ	0.374 ± 0.009 ^a^	0.416 ± 0.014 ^c^	0.088 ± 0.015 ^c^	0.073 ± 0.018 ^d^	0.016 ± 0.007 ^a^	0.017 ± 0.006 ^b^	5.500 ± 0.011 ^b^	4.294 ± 0.015 ^d^

DOC, dissolved organic carbon; POC, particulate organic carbon; PON, particulate organic nitrogen; POC/PON, particulate organic carbon to nitrogen ratios. Values within the same row with different letters represent significant difference (*p* < 0.05). Each value represents mean ± SD (*n* = 3).

**Table 3:** Central regions of EEM fluorescence attributed to different sources of organic matter compared with previous studies.

Traditional Peak	Ex/Em	Description	Probable Origin	Comparison with Previous Studies
Peak A	250(325)/425	Humic-like	Terrestrial	Humic-like C1: 320 (250)/422 [23] Humic-like C4: 325 (250)/416 [24]
Peak C	320-360/420-460	Humic-like	Terrestrial/autochthonous	Humic-like P8: <260 (355)/434 [25]
Peak M	290-310/370-420	Humic-like	microbial processing of organic matter	Humic-like P1: 310/414 [25]
Peak T	225(275)/330-340	Tryptophan-like	Autochthonous/amino acids, free or bound in proteins	Tryptophan-like, protein-like peak T: 225(275)/340 [26]
